# The surface structure of silver-coated gold nanocrystals and its influence on shape control

**DOI:** 10.1038/ncomms8664

**Published:** 2015-07-08

**Authors:** J. Daniel Padmos, Michelle L. Personick, Qing Tang, Paul N. Duchesne, De-en Jiang, Chad A. Mirkin, Peng Zhang

**Affiliations:** 1 Department of Chemistry, Dalhousie University, Halifax, Nova Scotia, Canada B3H 4R2.; 2 Department of Chemistry and International Institute for Nanotechnology, Northwestern University, Evanston, Illinois 60208, USA.; 3 Department of Chemistry, University of California, Riverside, California 92521, USA.; 4 School of Biomedical Engineering, Dalhousie University, Halifax, Nova Scotia, Canada B3H 4R2.

## Abstract

Understanding the surface structure of metal nanocrystals with specific facet indices is important due to its impact on controlling nanocrystal shape and functionality. However, this is particularly challenging for halide-adsorbed nanocrystals due to the difficulty in analysing interactions between metals and light halides (for example, chloride). Here we uncover the surface structures of chloride-adsorbed, silver-coated gold nanocrystals with {111}, {110}, {310} and {720} indexed facets by X-ray absorption spectroscopy and density functional theory modelling. The silver–chloride, silver–silver and silver–gold bonding structures are markedly different between the nanocrystal surfaces, and are sensitive to their formation mechanism and facet type. A unique approach of combining the density functional theory and experimental/simulated X-ray spectroscopy further verifies the surface structure models and identifies the previously indistinguishable valence state of silver atoms on the nanocrystal surfaces. Overall, this work elucidates the thus-far unknown chloride–metal nanocrystal surface structures and sheds light onto the halide-induced growth mechanism of anisotropic nanocrystals.

## Abstract (web summary)

Nanocrystal surface structure affects many properties but is tough to determine for halide-adsorbed materials. Here, the authors combine X-ray absorption measurements and computational modelling to elucidate the chloride metal surface structures for silver-coated gold nanocrystals with controlled shapes.

## Graphical abstract

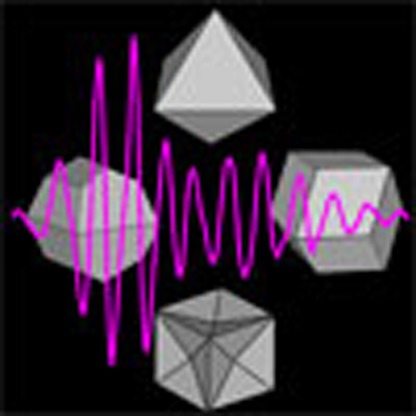

Metal nanocrystals (NCs) with well-defined shapes and facet indices are promising nanomaterials for many different applications due to the fascinating properties associated with their unique morphologies and surface structures^1^,^2^,^3^,^4^,^5^. Moreover, recent studies on silver (Ag)-assisted seeded growth with select halides have shown that gold (Au) NCs with unusual morphologies and high-index facets can be deliberately prepared in high yield^4^,^5^,^6^. The preparation of such Au NCs has demonstrated the correlation between Ag precursor concentration and Au NC facet index and shape. Most importantly, it has also identified the significance of the underpotential deposition (UPD) of Ag on the resulting rhombic dodecahedra with {110} facets, truncated ditetragonal prisms with {310} facets and concave cubes with {720} facets. The same method was also used to prepare Au octahedra with {111} facets, but their growth was found to be governed by a more typical thermodynamic formation process^6^. In addition, halide ions, such as the chlorides (Cl^−^) used in those syntheses, are an important factor governing the shape evolution of NCs prepared by the UPD method^5^. Briefly, halides have been shown to control the growth of specific facets on Au NC surfaces by (i) modifying the reduction potential and solubility of the Au^+^–halide complexes formed in solution and (ii) passivating the Au NC surface thereby changing the amount of Au surface available for the catalysed reduction of Au^+^ (ref. 5). Furthermore, in Ag-assisted Au NC syntheses, the resulting halide–Ag UPD layer can control specific facet growth by its stability on the Au NC surface, and the facet type produced is dependent on the type of halide used^7^. Although the synergistic roles of Ag and halides have been implicated in the formation mechanisms of the aforementioned NCs, their facet-specific surface structure is still largely unknown, despite being critically important to understanding their growth mechanisms and structure–property relationships. In particular, little is known about the halide portion of the structure, as halides are nearly invisible to traditional analytical techniques. Therefore, elucidating the surface structure of multicomponent systems, such as Cl^−^-adsorbed Ag-coated Au NCs, is a challenging yet worthwhile endeavour due to its impact on controlling NC shape and functionality.

Towards this end, X-ray absorption spectroscopy (XAS) can provide surface structure information regarding oxidation state, electronic structure and local coordination environment (including metal–metal and metal–adsorbate bonding) from the perspective of a given element^8^. Due to this element specificity, XAS is particularly useful in probing multicomponent surface structures, including Ag–Au bimetallic NCs^9^,^10^,^11^,^12^,^13^,^14^. Although typically extracting information regarding surface-bonding configurations from XAS is limited to small nanoparticles (that is, <5 nm), the signals from the more numerous non-surface atoms in larger nanoparticles outweigh those from the surface atoms. However, in the case of the relatively large NCs described herein (that is, >50 nm), their surfaces are covered by a thin layer of Ag that can be isolated using element-specific XAS, thus allowing their surface structures to be determined. In addition, density functional theory (DFT) can be used to model the coordination environments of these NC surfaces, as their large sizes permit their treatment as bulk surfaces. DFT has been previously used to calculate adsorbate interactions with low-index bulk metal surfaces ({111}, {110}, {100} and so on)^15^,^16^,^17^,^18^; however, higher-index surfaces, let alone those on NCs, are less commonly prepared and have not been correspondingly studied with DFT.

In this study, we implement element-specific XAS to reveal the atomic surface structures of Cl^−^-adsorbed Ag-coated Au NCs with {111}, {110}, {310} and {720} surface facets. In this way, we identify key differences between non-UPD and UPD NC surface coordination and bonding, as well as characterize the facet-index-dependent Ag–Cl bonding between all of the NCs. We then use DFT structural optimization calculations to generate a more complete description of the NC surfaces, in particular the Cl^−^ adsorbates on Ag. Moreover, we systematically explore the effect of Cl^−^ adsorption on each NC surface by XAS simulations, and those results are used in conjunction with experimental XAS to both identify the Ag valence state on the NC surfaces and further confirm the Ag–Cl bonding environment. This combination of experimental/simulated XAS and DFT modelling demonstrates the ability to comprehensively study the surface structure of shape and facet-index-controlled bimetallic NCs.

## Results

### Nanocrystal surface structure

**Figure 1:**
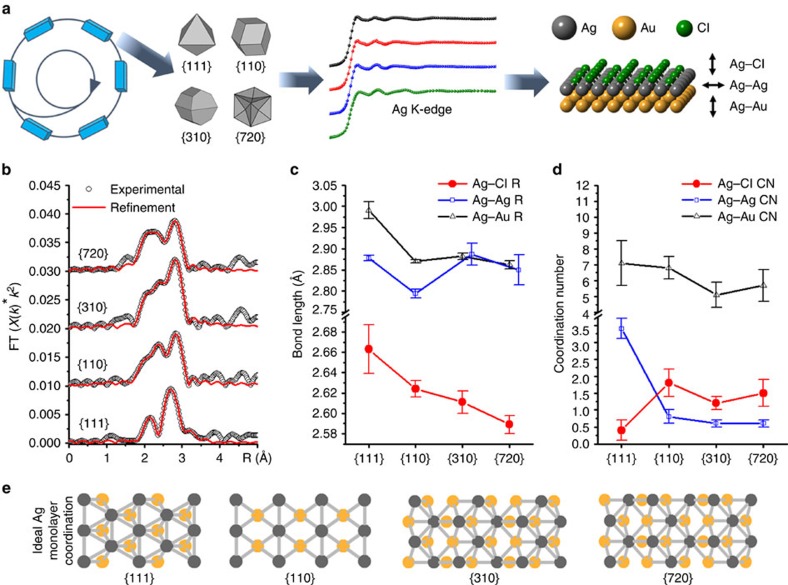
Surface structure of Ag-covered Au NCs from synchrotron Ag K-edge XAS. (**a**) Schematic illustration of synchrotron X-rays used to obtain Ag K-edge EXAFS spectra from the Ag on the NC surfaces, from which three-layer Ag–Cl, Ag–Ag and Ag–Au surface structures were determined. (**b**) Experimental EXAFS *R*-space spectra of the Au NCs with refinements based on theoretical bonding paths generated by FEFF. The refinements were conducted over *R*-space ranges of 1.7–3.3 Å, hence the disagreement of the refinements to the experimental data outside of that range. (**c**) Experimentally determined Ag–Cl, Ag–Au and Ag–Ag bond lengths and (**d**) coordination numbers of each NC surface. The error bars in c and **d** represent uncertainties in the refinement, and details of their calculation can be found in the Methods section. (**e**) Top-down view of ideal monolayer Ag coverage on Au with {111}, {110}, {310} and {720} facets, giving average Ag–Ag/Ag–Au coordination numbers of 6/3, 2/5, 2/5 and 3/4.8, respectively.

The preparation and general characterization of octahedra with {111} facets, rhombic dodecahedra with {110} facets, truncated ditetragonal prisms with {310} facets and concave cubes with {720} facets have been published elsewhere^6^. Briefly, their specific facets were identified by a combination of electron diffraction and facet-angle measurements using scanning electron microscopy, and were found to have approximate edge lengths between 70 and 120 nm (refs 6, 19, 20). The Ag coverage on each surface was identified by a combination of X-ray photoelectron spectroscopy and inductively coupled plasma spectroscopy, and each NC type exhibited slightly lower than full monolayer (ML) Ag coverages (60–80%)^6^. In the work presented here, synchrotron Ag K-edge extended X-ray absorption fine-structure (EXAFS) experiments were used to elucidate a three-component bonding structure composed of Ag–Cl, Ag–Au and Ag–Ag for each NC surface (schematically illustrated in Fig. 1a). The validity of this three-component structure is supported by the high-quality EXAFS refinements shown in Fig. 1b (see Supplementary Table 1 for the EXAFS results table and Supplementary Figs 1–4 for the refinement components and residuals). The most notable result from the EXAFS bond length analysis (Fig. 1c) is that the {111} Ag–Cl bond (2.66±0.02 Å) is longer than those of the {110} (2.62±0.01 Å), {310} (2.61±0.01 Å) and {720} (2.58±0.01 Å) surfaces. Likewise, the Ag–Au bond length of the {111} sample (2.99±0.03 Å) is longer than those of the {110} (2.87±0.01 Å), {310} (2.88±0.01 Å) and {720} (2.86±0.01 Å) samples. These two findings indicate the remarkable difference in adsorbate and alloy bonding between the non-UPD and UPD NCs (that is, {111} versus {110}, {310} and {720}), and illustrate the effectiveness of the UPD mechanism in inducing stronger Ag–Cl and Ag–Au interactions (that is, shorter bond lengths). Furthermore, the Ag–Cl bond lengths in the NCs are shown to be facet-index dependent, suggesting that the Ag–Cl bonding is a key component in stabilizing the increasing surface energies of the NCs (that is, *γ*_111_<*γ*_110_<*γ*_310_<*γ*_720_ (ref. 21)). On the other hand, there is no discernable trend in the Ag–Au bond length between the UPD samples, and therefore the Ag–Au bonding in these structures is likely more dependent on the UPD mechanism rather than facet-specific bonding. Moreover, the Ag–Ag bond lengths do not demonstrate any clear trend, which may be a result of interplay between facet-specific bonding and the UPD mechanism.

The average coordination numbers (CNs) of each NC surface were revealed from the EXAFS results and plotted in Fig. 1d. To assist in the interpretation of the CNs, ideal monolayer models of Ag on Au (Fig. 1e) were used to calculate average Ag–Ag/Ag–Au CNs, yielding 6/3 for {111}, 2/5 for {110}, 2/5 for {310} and 3/4.8 for {720}. To compare the experimentally observed CNs to the ideal monolayer CNs, it is first useful to note that NC surfaces are commonly found to have under-coordinated surface sites (edges, corners, defects and so on)^22^. In addition, the slightly less-than-monolayer coverages of Ag on the NCs could contribute to under-coordinated Ag. The under-coordinated Ag on these NC surfaces will greatly influence their Ag–Ag CNs, and could explain why the Ag–Ag CNs are lower than the ideal (that is, 3.4±0.3 versus 6 for {111}, 0.8±0.1 and 0.6±0.1 versus 2 for {110} and {310}, respectively, and 0.6±0.1 versus 3 for {720}). However, the subsurface Au (the main component of the NCs) will be less influenced by Ag under-coordination, and therefore the Ag–Au CN is a more reliable parameter to evaluate the surface structure in these cases. Taking this into account, the Ag–Au CNs of the two high-index surfaces (4.9±0.8 for {310} and 5.7±0.9 for {720}) are consistent with the ideal monolayer models (Ag–Au CN of 5 and 4.8). On the other hand, the {110} shows a slightly higher Ag–Au CN (6.8±0.7) than the ideal Ag–Au CN of 5, indicating that the Ag is perhaps present both in and below the top-most surface layer. However, the {110} Ag–Cl CN of 1.8±0.4 is similar to 1.2±0.2 for {310} and 1.5±0.4 for {720}, indicating that the Ag is mostly on the surface and therefore more consistent with a monolayer model. In contrast to the UPD NCs, the {111} Ag–Au CN of 7.1±1.4 is significantly higher than the ideal CN (that is, 3). This is consistent with interdiffused Ag on Au–Ag core-shell NCs^11^, and supports the presence of Ag below the surface of the {111} NCs. In addition, the Ag–Cl CN is significantly lower for the {111} NCs (that is, 0.4±0.2) compared with that of the three UPD NCs with Ag monolayer-type structures. This low Ag–Cl CN further supports the notion that there is a significant amount of Ag below the {111} NC surface. Conversely, the higher Ag–Cl CNs of the {110}, {310} and {720} NCs support that the Ag is predominantly on their surfaces, and also demonstrates the increased Cl^−^ adsorption from the UPD mechanism^23^. Collectively, the Ag–Ag, Ag–Au and Ag–Cl CN results suggest that the Ag atoms in the {111} NC surface are both in and below the top-most metal layer, whereas the {110}, {310} and {720} NC surfaces are more distinct Ag monolayer-type structures.

### Density functional theory modelling

**Figure 2:**
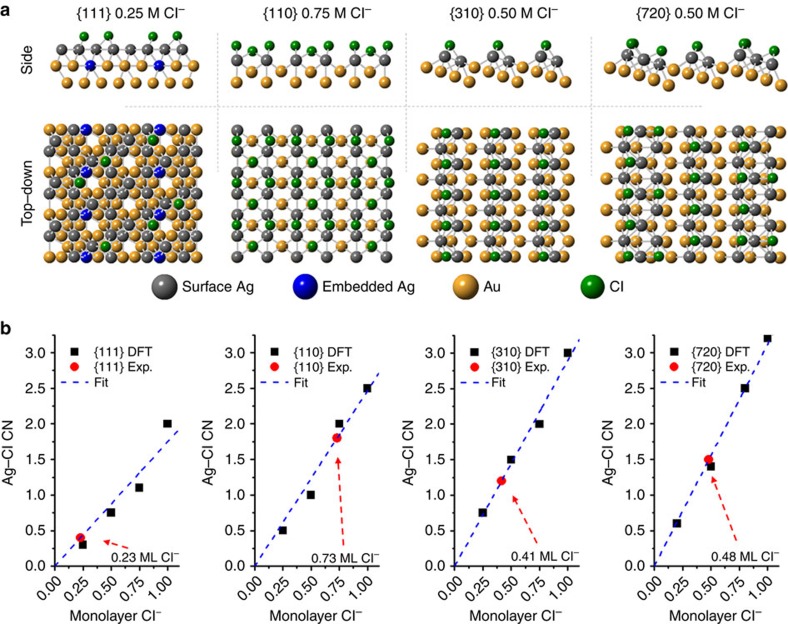
DFT model-optimization results. (**a**) Side and top-down views of DFT-optimized structures with Cl^−^ coverage of 0.25 ML Cl^−^, 0.75 ML Cl^−^, 0.50 ML Cl^−^ and 0.50 ML Cl^−^ for the {111}, {110}, {310} and {720} NC surfaces, respectively. (**b**) Calibration curves of Cl^−^ coverage versus Ag–Cl CN for each calculated surface, and the corresponding experimental Cl^−^ coverage.

To confirm the experimental EXAFS coordination trends and provide more detailed information about the Ag–Cl, we carried out DFT structural optimization calculations for the {111}, {110}, {310} and {720} surfaces (see models in Fig. 2a). In these structural optimizations, we considered the amount of Cl^−^ coverage and compared 0, 0.25, 0.50, 0.75 and 1.0 (full) Cl^−^ monolayer (ML) coverages on each surface type (20 DFT models in total, see Supplementary Figs 5–9). Remarkably, the {111} DFT model (see Fig. 2a top left) was in good agreement with an embedded Ag configuration (that is, Ag below and in top-most layer), while the {110}, {310} and {720} DFT models showed more distinct Ag monolayer-type structures (Fig. 2a). These DFT-optimized structures were also in excellent agreement with the EXAFS CN results (see Supplementary Table 2 for further comparison). Moreover, the DFT results were used to determine actual Cl^−^ coverages on the NC surfaces by plotting Ag–Cl CN versus Cl^−^ coverage for each DFT-optimized surface model (Fig. 2b). The high-index UPD NCs were found to have similar Cl^−^ coverages of 0.41 ML for {310} and 0.48 ML for {720}. Interestingly, the higher Cl^−^ coverage for the {110} UPD NCs (0.73 ML) is consistent with a higher degree of Cl^−^ interaction to stabilize the surface, as it has the lowest number of surface Ag atoms per unit area of Au^6^. In contrast, the {111} Cl^−^ adsorption was much lower than the others (0.23 ML), again highlighting its difference from the UPD NCs and affirming the notion that it is stabilized by a different mechanism.

### Validation of surface structure and identification of silver valence state

**Figure 3:**
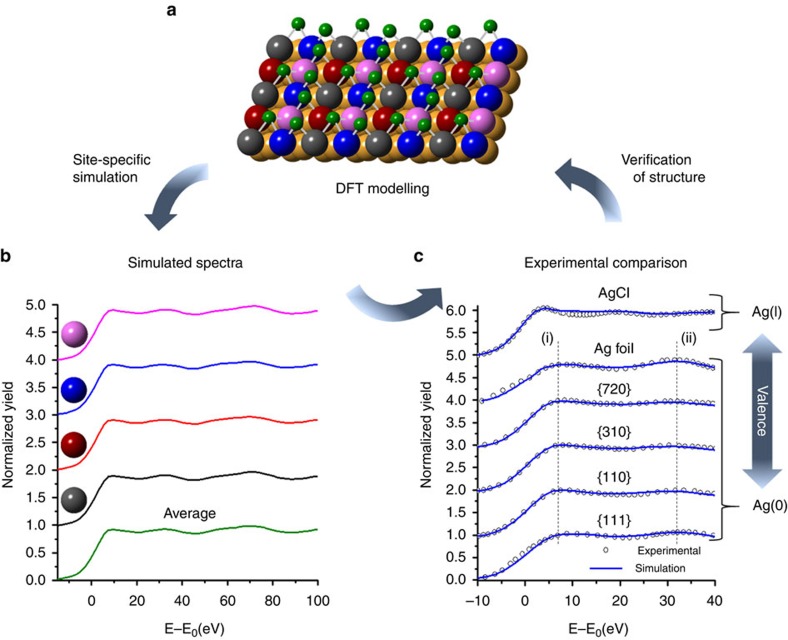
Combined approach of three techniques for validation of surface structure and identification of Ag valence state. (**a**) DFT-optimized model of the {110} NC surface showing the four different Ag sites shown in different colours. (**b**) Site-specific XANES spectra of the {110} surface were simulated by the FEFF program and then averaged. The simulated XANES spectra for the other surfaces are shown in the Supplementary Figs 10–13. (**c**) Averaged simulated XANES spectra were compared with experimental XANES spectra to verify the DFT models, as well as compared with reference materials to determine the valence state of Ag on the NC surfaces.

To verify the surface structure models deduced above, we employed a combined approach using DFT-optimized models (Fig. 3a), site-specific simulations of X-ray absorption near edge structure (XANES) (Fig. 3b), and experimental XANES (Fig. 3c). In this way, site-specific Ag K-edge XANES simulations were carried out with the FEFF program using the coordinates provided by the DFT-optimized models of the proposed Cl^−^ coverages (see Supplementary Figs 10–13). The site-specific simulations of each surface were averaged and overlayed with experimental data (Fig. 3c) and in each case were found to fit well with the simulations, thus verifying the proposed DFT models (Fig. 2a). Furthermore, the spectral feature (i) in Fig. 3c for each surface matches with the reference Ag foil, indicating that the surfaces are of a similar valence state (that is, zero-valent). The later oscillatory feature (ii) in Fig. 3c of all of the surfaces also lines up with the reference Ag foil, denoting similar metal–metal interactions, although it is less intense because of the lower backscattering amplitude from fewer Ag neighbours in the NC surfaces. In contrast, the NC spectral features are much different from the AgCl reference, which rules out the presence of ionic Ag and highlights the more extensive halide–metal bonding nature of the NC surfaces. We also carried out Ag L_3_-edge XANES experiments on each NC sample to further confirm the oxidation state of the Ag on the NC surfaces (see Supplementary Fig. 14). The NCs all exhibited similar features to the Ag(0) reference and showed the typical reduction in the first peak of the XANES due to nanosize and alloying effects^24^,^25^, thereby confirming the metallic oxidation state of the Ag on each surface.

### Effect of chloride adsorption on the nanocrystal surfaces

**Figure 4:**
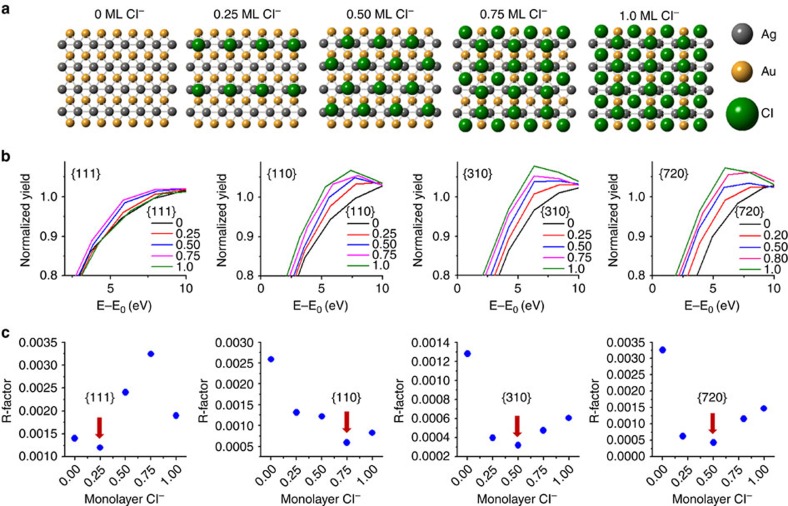
Simulated Ag K-edge XANES spectra and linear combination fitting results. (**a**) Representative illustrations of the {110} DFT-optimized models with different Cl^−^ coverages. The Cl^−^ was enlarged to more clearly see the different coverages. (**b**) Averaged XANES simulation from each DFT-modelled surface with 0, 0.25, 0.50, 0.75 and 1.0 ML Cl^−^ coverages. The averaged spectra were normalized to their absorption energies and used as individual standards to conduct a linear combination fit of the experimental data. The closeness of the averaged model spectra to the experimental spectra are represented by the *R*-factors shown in **c**. The closest fit is characterized by the lowest *R*-factor, also indicated by the red arrow for each surface.

Since the XANES region of metal K-edges are sensitive to both ligand adsorption and coordination^26^, further Ag K-edge XANES simulations were carried out to evaluate the effect of different amounts of Cl^−^ adsorption on each DFT-modelled surface (see Supplementary Figs 15–18 for the complete set of 131 simulations). The simulations for each surface with the five different Cl^−^ coverages (Fig. 4a shows the {110} surface for illustration) were averaged and a significant change in the position and intensity of the first XANES peak in proportion to the amount of Cl^−^ adsorbed was observed (Fig. 4b). The averaged XANES were then used to conduct a linear combination fit of the experimental data of the corresponding NC surface and the best fitting model was identified by the lowest *R*-factor (Fig. 4c)^27^,^28^. Remarkably, the Cl^−^ coverage results from the linear combination fits are in excellent agreement with the findings shown in Figs 2 and 3, and further support the NC surface structures presented in this work.

## Discussion

In this work, we uncovered the multicomponent surface structures of Cl^−^-adsorbed, Ag-coated {111}, {110}, {310} and {720} Au NCs. These NCs represent excellent systems to elucidate the unique properties of NCs derived from specific synthetic approaches (for example, UPD versus non-UPD) and specific surface crystallography (for example, low versus high index). Our results can also help to clarify a number of fundamental concepts related to shape-controlled anisotropic NCs. First, the surface structures of the NCs were identified at the atomic scale and shown to be composed of Ag–Cl, Ag–Ag and Ag–Au bonding. Interestingly, the NC surfaces comprised metallic Ag, demonstrates the ability of these NCs to maintain the metallic nature of the less-noble surface metal (that is, Ag). This finding is in contrast to the typical surface oxidation that occurs at other Ag NC surfaces. Second, the significant differences between non-UPD and UPD NC surface structure were revealed in this work. In particular, the non-UPD NCs ({111}) exhibited Ag atoms embedded within the subsurface Au, whereas the UPD NCs ({110}, {310} and {720}) showed more distinct Ag monolayer-type structures on top of Au. This finding alone may have a substantial impact on the surface engineering of bimetallic NCs, as the UPD method may represent an important strategy to prevent the commonly observed metal interdiffusion at NC surfaces^11^. In addition, the Ag–Au alloy bond of the non-UPD NCs was significantly longer than those of the UPD NCs, implying that the UPD mechanism produced a stronger alloying interaction (that is, shorter alloy bonding) at the surface of the NCs. Third, and most importantly, this work sheds light on the still-debated halide-induced formation mechanisms of shape-controlled Au NCs^5^ by providing a detailed picture of the surface halide–metal bonding. So far, the special stabilization of Ag by Cl^−^ adsorbates has been proposed as an explanation for the formation of a wider variety of Au NC shapes in the presence of Cl^−^ than in the presence of other halides, such as Br^−^ and I^−^ (ref. 29). The four NCs studied in this work exhibited surface-index-dependent Ag–Cl bond lengths and coverage, and such remarkable flexibility of the Ag–Cl bonding on the NC surfaces helps to explain this special Cl^−^ stabilization mechanism and particular NC growth. Finally, our work also demonstrates the usefulness of combining EXAFS, DFT modelling and experimental/simulated XANES to reliably uncover the surface structure of shape and facet-index-controlled bimetallic NCs. The potential of this technique towards more in-depth studies of bimetallic NC formation mechanisms is enormous, given the ability of *in situ* and liquid/solid phase element-specific XAS measurements^30^.

## Methods

### Nanocrystal synthesis

The synthesis and characterization methods of the NC samples has been previously published^6^. Briefly, cetyltrimethylammonium chloride-stabilized seed Au NCs were mixed with a growth solution containing cetyltrimethylammonium chloride, hydrochloric acid, ascorbic acid, hydrogen tetrachloroaurate (HAuCl_4_) and differing amounts of silver nitrate (AgNO_3_). The concentration of AgNO_3_ has been shown to determine the resulting shape and surface index and samples prepared with 1, 10, 40 and 100 μM AgNO_3_ produced {111} octahedra, {110} faceted rhombic dodecahedra, {310} faceted truncated ditetragonal prisms and {720} faceted concave cubes, respectively.

### X-ray absorption spectroscopy

After preparation, the samples were freeze-dried and packed into kapton film pouches to conduct XAS experiments at the Advanced Photon Source (BM-20 beamline, Argonne National Laboratory, Argonne, IL, USA) or the Canadian Light Source (SXRMB beamline, Saskatoon, SK, Canada). For Ag K-edge XAS measurements, the samples were placed in a cryostatic sample holder at 50 K while the Ag K-edge XAS measurements were collected using a 32-element Ge fluorescence detector; XAS data for Ag foil was collected simultaneously using standard gas-ionization chamber detectors. This low-temperature fluorescence XAS method was used to compensate for the dilute concentration of Ag in the sample, and to reduce the dynamic thermal disorder in the sample^31^. For Ag L_3_-edge XAS, the freeze-dried samples were affixed to double-sided carbon tape on a sample holder and then placed in the sample chamber and allowed to reach an ultra-high vacuum atmosphere. The Ag L_3_-edge XANES spectra were collected with a four-element Silicon drift detector.

### EXAFS spectra refinement

All of the XAS spectra were processed with the WinXAS or Ifeffit^27^,^28^ software packages. The raw EXAFS data (see Supplementary Fig. 19) were converted to *k*-spaces (see Supplementary Fig. 20) and then Fourier-transformed into *R*-spaces (see Fig. 1a and Supplementary Figs 1–4) using a range of *k*=2.5–12.7, which was chosen by the overall data quality of the samples. The *R*-spaces were then refined with WinXAS using bonding paths from an *ab initio* simulation of Cl^−^-adsorbed Ag on the Au{111} model generated using the FEFF program (version 8.2)^32^,^33^. The refinements calculated structural information such as CN, bond length and Debye–Waller factors (*σ*^2^) along with the energy-shift parameter, Δ*E*_0_, which helps account for refinement assumptions. Given the composition of the samples, it was surmised that Ag–Cl, Ag–O, Ag–N, Ag–Ag and Ag–Au were bonding paths, as those theoretical phase amplitudes generated by FEFF are contained within the *R*-space region of around 2–3 Å (without phase correction). However, tentative refinements of Ag–N and Ag–O paths produced entirely unphysical results; thus, they were not considered in any further refinements. The smaller peaks under 1.5 Å for the samples are most likely too short to be attributed to nearest neighbour backscattering and may be an artefact of low-frequency noise in the fluorescence data. Therefore, all of the *R*-spaces underwent refinements with Ag–Cl, Ag–Ag and Ag–Au bonding paths within the *R*-space range of 1.7–3.3 Å. To reduce the number of free running parameters to number of independent points, the Δ*E*_0_ values for all of the paths, and the *σ*^2^ values for the two metal–metal paths were correlated. The Δ*E*_0_ values account for the phase differences of the experimental data to the theoretical FEFF input data. These values are often correlated in EXAFS refinements, given the assumption that each path will have the same phase differences if the same model was used in the input files. In our case, the same model was used and therefore we can correlate them to reduce the number of variables. In addition, the *σ*^2^-values were correlated because Ag and Au have very similar lattice constants and bonding, therefore the disorder in their bonds should be similar. It should be noted that the {111} spectrum could not be refined with correlated *σ*^2^, which is likely a result of the very different bonding arrangement of the Ag in that sample (for example, higher degree of alloying). For each refinement, WinXAS calculated the number of free parameters to be 8 (except for {111} with 9 free parameters) and the number of independent points to be 13. The uncertainties reported were calculated from off-diagonal elements of the correlation matrix of each fit, weighted by the square root of the reduced *χ*^2^-value, taking into account the experimental noise for each *R*-space spectrum from 15 to 25 Å (ref. 34).

### Density functional theory modelling

The DFT computations were performed with a frozen-core plane-wave pseudopotential approach using the Vienna *ab initio* simulation package^35^. Since the NCs were relatively large, periodic slab surface models with different indices represented the corresponding facets of the NCs. A 4 × 4 × 1, 2 × 2 × 1, 2 × 2 × 1 and 2 × 1 × 1 supercell size was used to simulate the {111}, {110}, {310} and {720} surfaces, which contained about 80/16, 28/4, 20/4 and 58/10 Au/Ag atoms, respectively. Among these surfaces, {111} surface has a planar shape, while {110}, {310} and {720} surfaces have step-like shapes. The Au {111}, {110}, {310} and {720} periodic slab surfaces were modelled with 6, 7, 10 and 13 layers of Au atoms, respectively, and are within a thickness of 1–2 nm, to provide qualitatively reasonable predictions on the NC core behaviour. The Cl^−^ anion was modelled by placing Cl atoms on the slab surfaces, letting the electronic density converge and then determining the partial atomic charges on the Cl atoms. The full 1.0-ML Cl^−^ coverage for each surface was created by the following: for the {111} surface, about ¼ Ag diffuse into the Au layer, and the vacant surface Ag sites were then occupied by Cl, while the remaining ¾ of surface Ag atoms are coordinated to adsorbed Cl^−^ in the 3:1 ratio. For the {310} and {720} surfaces, full coverage was also created by coordinating Cl^−^ in a 3:1 ratio, while for the {110} surface, a 4:1 ratio was used. On the basis of the full coverage model for each surface, other coverage models were constructed (for example, 25, 50 and 75%) by taking out either the substituted Cl^−^ or adsorbed Cl randomly, and then used a low-lying energy model for further structural analysis. During the computations, pseudopotentials with 5d^10^6s^1^, 4d^10^5s^1^ and 3s^2^3p^5^ valence electron configurations were used for Au, Ag and Cl atoms, respectively, and the scalar-relativistic effect was included in the pseudopotential for Au. Since the surfaces contained strong covalent Ag–Cl and Ag–Au bonds, no correction for the long-range aurophilic interactions was implemented. The ion–electron interaction was described with the projector-augmented wave method^36^. Electron-exchange correlation was represented by the generalized gradient approximation functionals from Perdew, Burke and Ernzerhof^37^. A cutoff energy of 450 eV was used for the plane-wave basis set. The Brillouin zone was sampled using a 4 × 4 × 1 Monkhorst-Pack *k*-point mesh. The convergence threshold for structural optimization was set to be 0.02 eV Å^−1^ in interatomic force. The thickness of the vacuum layer was set to be 12 Å, which is large enough to ensure decoupling between neighbouring slabs, as the further increase of the vacuum thickness leads to a small energy change of <0.01 eV. During optimization, the top three, two, four and six layers of the respective {111}, {110}, {310} and {720} Au surfaces were allowed to relax together with the surface Ag layer and adsorbed Cl atoms, while the bottom layers were kept at their bulk positions. The resulting atomic coordinates from the DFT computations were obtained for each surface and modelled with the Crystal Maker program (version 9.0.2).

### XANES simulations and linear combination fitting

Simulated Ag K-edge XANES spectra were calculated using the FEFF program with atomic coordinates obtained from the DFT-optimized structures. The coordinates for the bulk models were generated from their respective symmetries and space groups. For each simulation, a full multiple scattering (FMS) diameter of 30 Å and a self-consistent field (SCF) radius of 6 Å was used for each individual Ag site, which equated to ∼100 and 30 atoms for the FMS and SCF calculations, respectively. An amplitude reduction factor (*S*_0_^2^) of 0.95 was used to render the results directly comparable to the experimental data. The linear combination fitting of the Ag K-edge XANES spectra were conducted with Athena, part of the Iffeffit software package^27^,^28^. In this way, the experimental XANES of each NC sample was individually fit with the corresponding average of the simulated XANES spectra for each coverage model. The linear combination fit was conducted within a range of −20 to 30 eV of the *E*_0_-normalized spectra. The resulting *R*-factor, which represents the goodness of fit, was recorded for each coverage model.

## Additional information

**How to cite this article:** Padmos, J. D. *et al*. The surface structure of silver-coated gold nanocrystals and its influence on shape control. *Nat. Commun.* 6:7664 doi: 10.1038/ncomms8664 (2015).

## Supplementary Material

Supplementary Information Supplementary Figures 1-20, Supplementary Tables 1-2.
