# Supplementary material for: The surface structure of silver-coated gold nanocrystals and its influence on shape control
Source: Nat Commun. 2015 Jul 8;6:7664. doi: 10.1038/ncomms8664 (PMC4510708; doi:10.1038/ncomms8664)
Supplement: Supplementary Information — Supplementary Figures 1-20, Supplementary Tables 1-2. [file ncomms8664-s1.pdf]

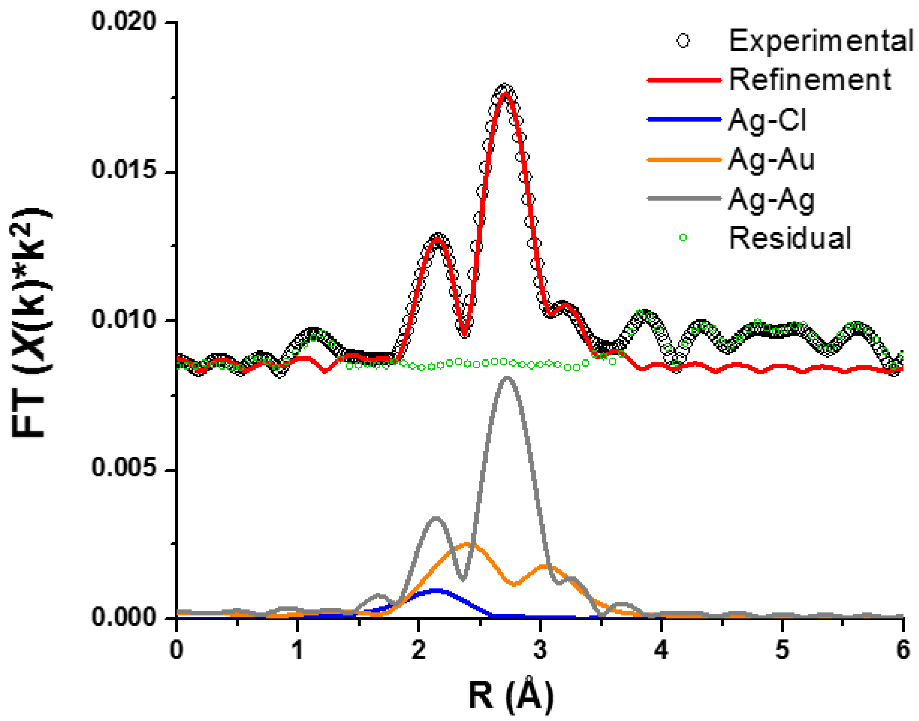


**Supplementary Figure 1. EXAFS refinement spectra of the {111} NCs.** The Fourier-transformed EXAFS were refined with Ag-Cl, Ag-Au, and Ag-Ag paths (shown individually for clarity) and contribute to the overall refinement (red) of the experimental data (black circles). The refinement residual is also given which shows the good agreement between the refinement range of 1.7-3.3 Å.

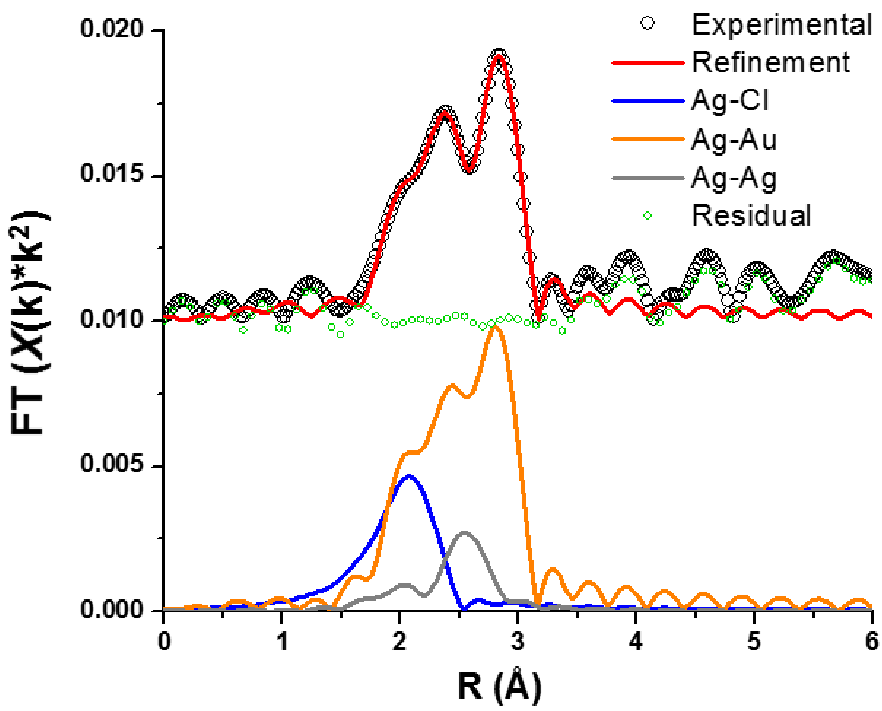


**Supplementary Figure 2. EXAFS refinement spectra of the {110} NCs.** The Fourier-transformed EXAFS were refined with Ag-Cl, Ag-Au, and Ag-Ag paths (shown individually for clarity) and contribute to the overall refinement (red) of the experimental data (black circles). The refinement residual is also given which shows the good agreement between the refinement range of 1.7-3.3 Å.

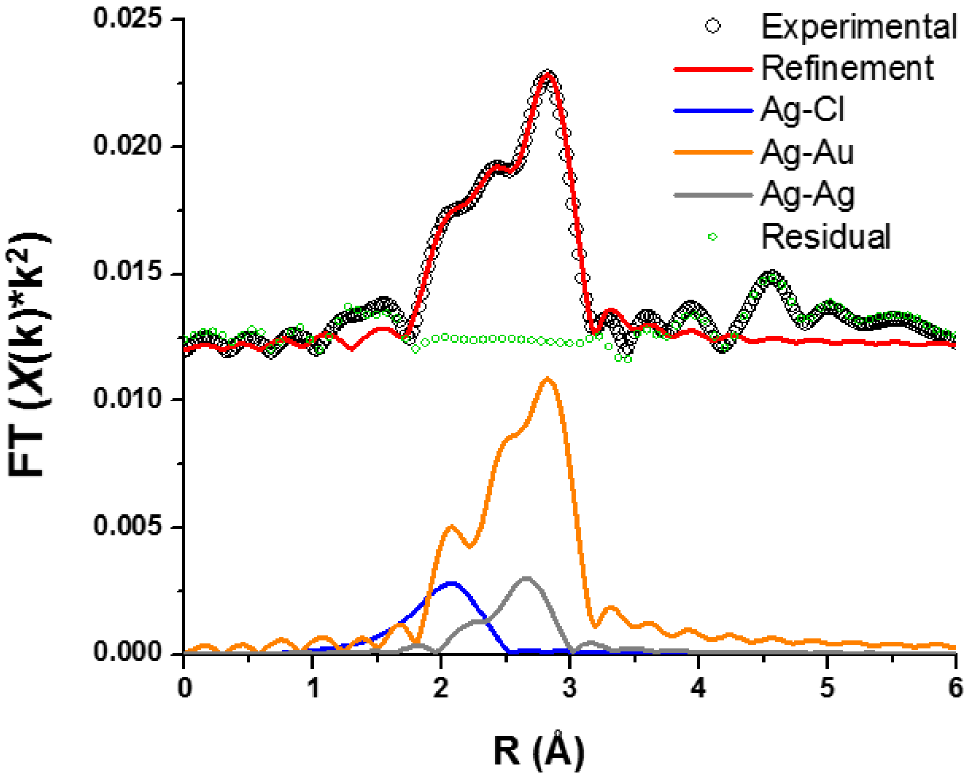


**Supplementary Figure 3. EXAFS refinement spectra of the {310} NCs.** The Fourier-transformed EXAFS were refined with Ag-Cl, Ag-Au, and Ag-Ag paths (shown individually for clarity) and contribute to the overall refinement (red) of the experimental data (black circles). The refinement residual is also given which shows the good agreement between the refinement range of 1.7-3.3 Å.

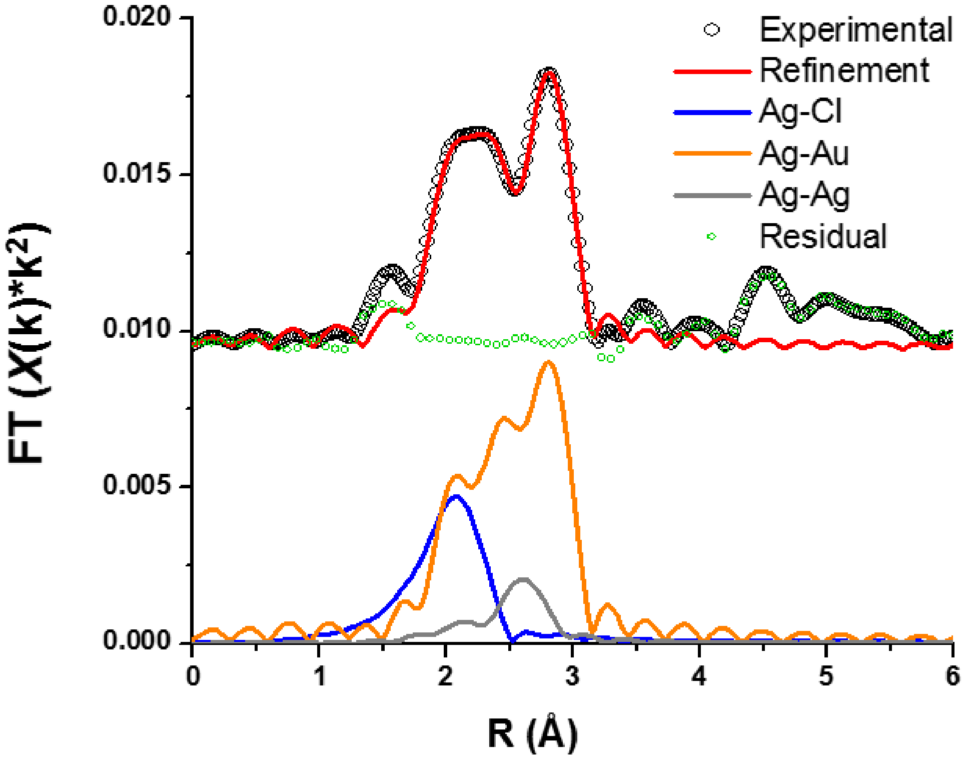


**Supplementary Figure 4. EXAFS refinement spectra of the {720} NCs.** The Fourier-transformed EXAFS were refined with Ag-Cl, Ag-Au, and Ag-Ag paths (shown individually for clarity) and contribute to the overall refinement (red) of the experimental data (black circles). The refinement residual is also given which shows the good agreement between the refinement range of 1.7-3.3 Å.

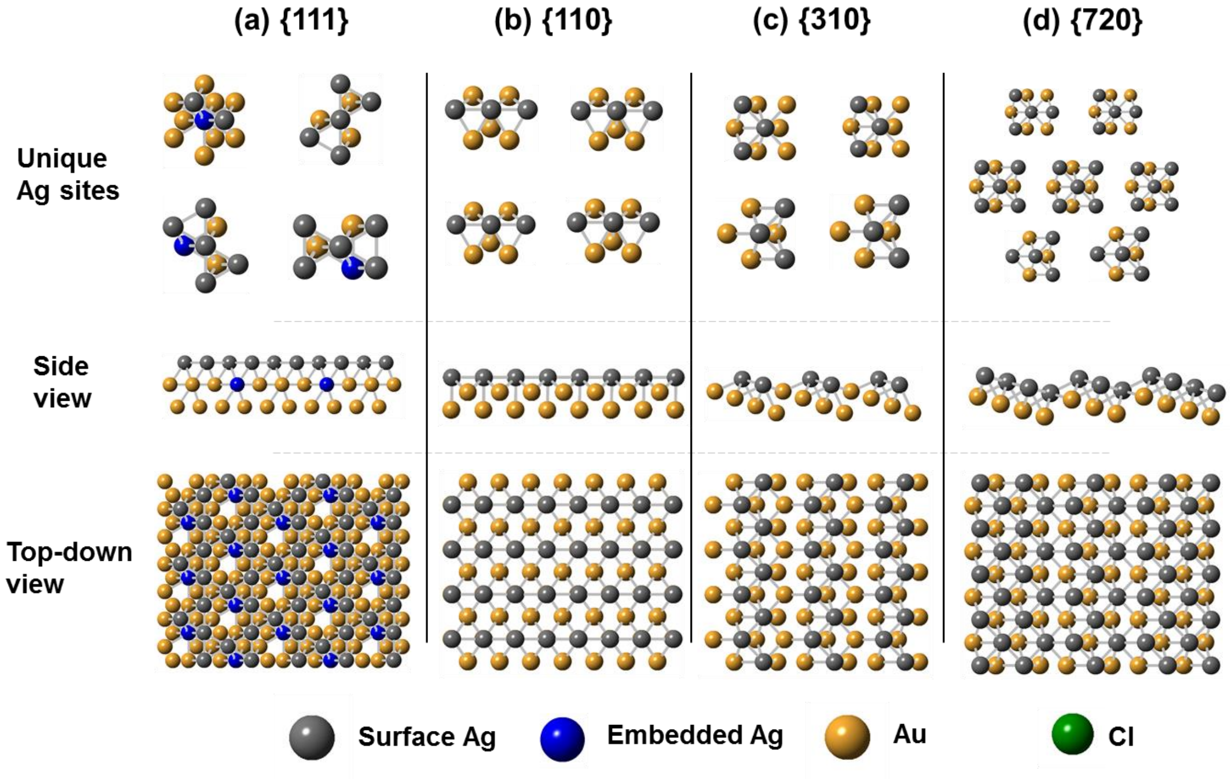


**Supplementary Figure 5. 0 ML $Cl^-$ DFT models.** The DFT optimized atomic coordinates of the (a) {111}, (b) {110}, (c) {310}, (d) {720} NC surfaces as modelled by Crystal Maker®. The unique Ag sites help show their individual coordination while the side and top-down views of each NC surface show overall perspective.

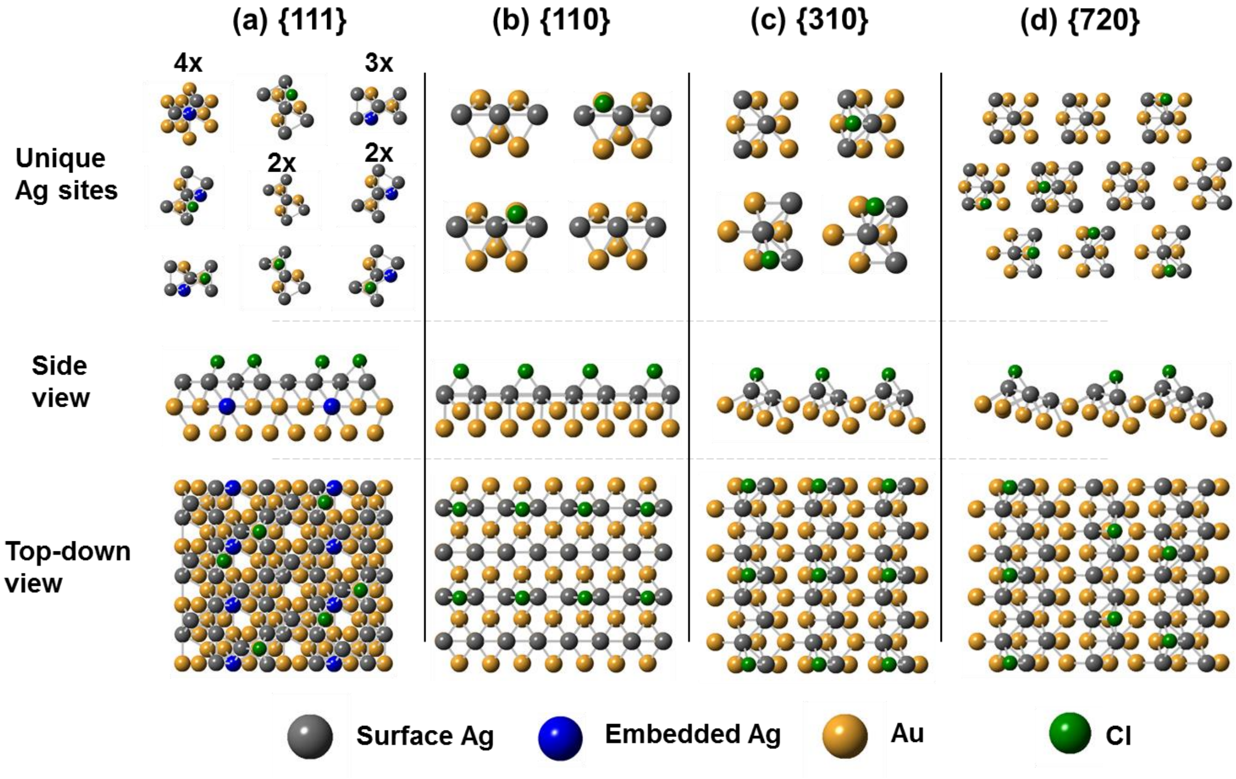


**Supplementary Figure 6. 0.25 ML $Cl^-$ DFT models.** The DFT optimized atomic coordinates of the (a) {111}, (b) {110}, (c) {310}, (d) {720} (0.20 ML) NC surfaces as modelled by Crystal Maker®. The unique Ag sites help show their individual coordination while the side and top-down views of each NC surface show overall perspective.

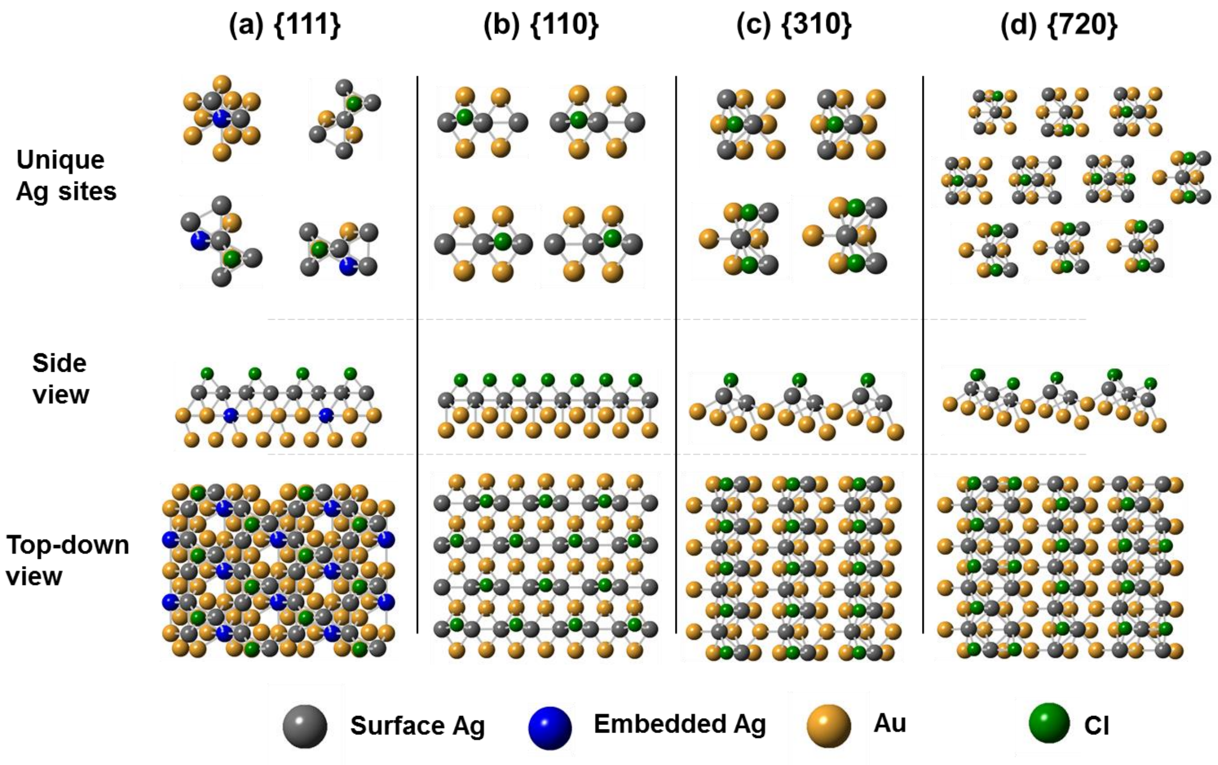


**Supplementary Figure 7. 0.50 ML $Cl^-$ DFT models.** The DFT optimized atomic coordinates of the (a) {111}, (b) {110}, (c) {310}, (d) {720} NC surfaces as modelled by Crystal Maker®. The unique Ag sites help show their individual coordination while the side and top-down views of each NC surface show overall perspective.

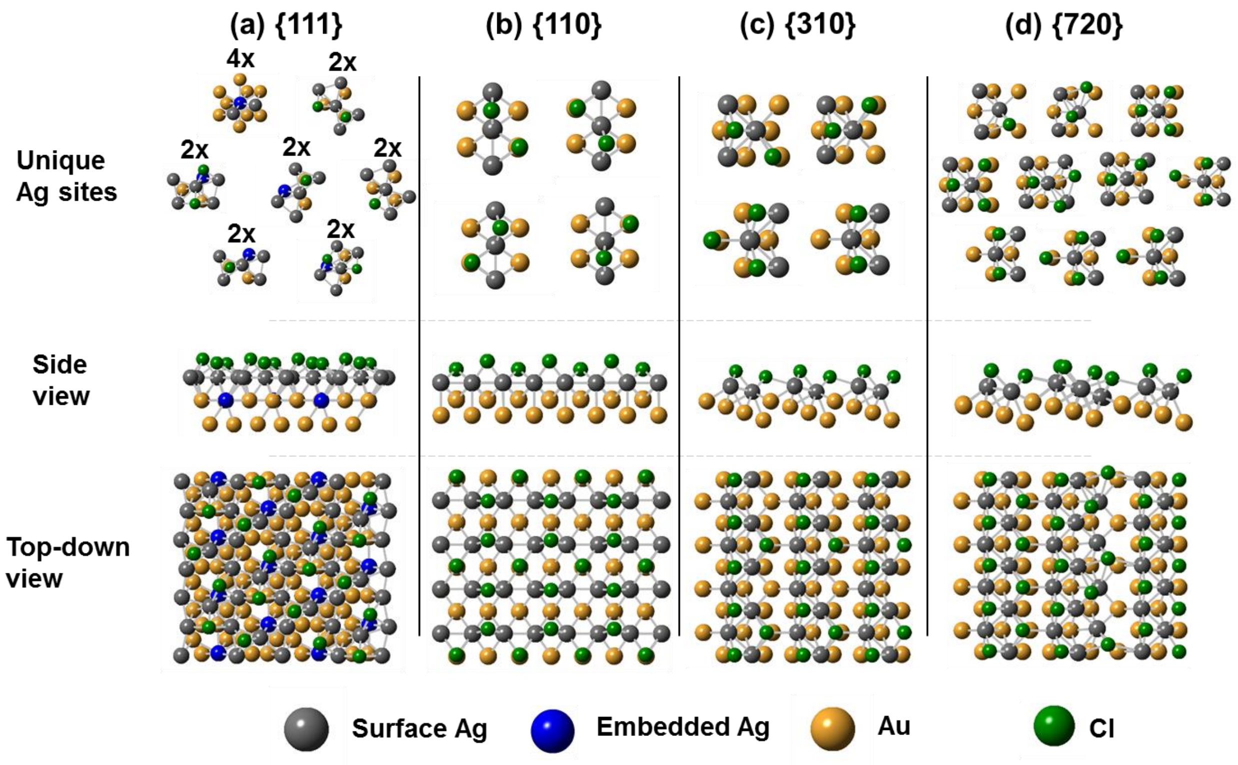


**Supplementary Figure 8. 0.75 ML Cl$^-$ DFT models.** The DFT optimized atomic coordinates of the (a) {111}, (b) {110}, (c) {310}, (d) {720} (0.80 ML) NC surfaces as modelled by Crystal Maker®. The unique Ag sites help show their individual coordination while the side and top-down views of each NC surface show overall perspective.

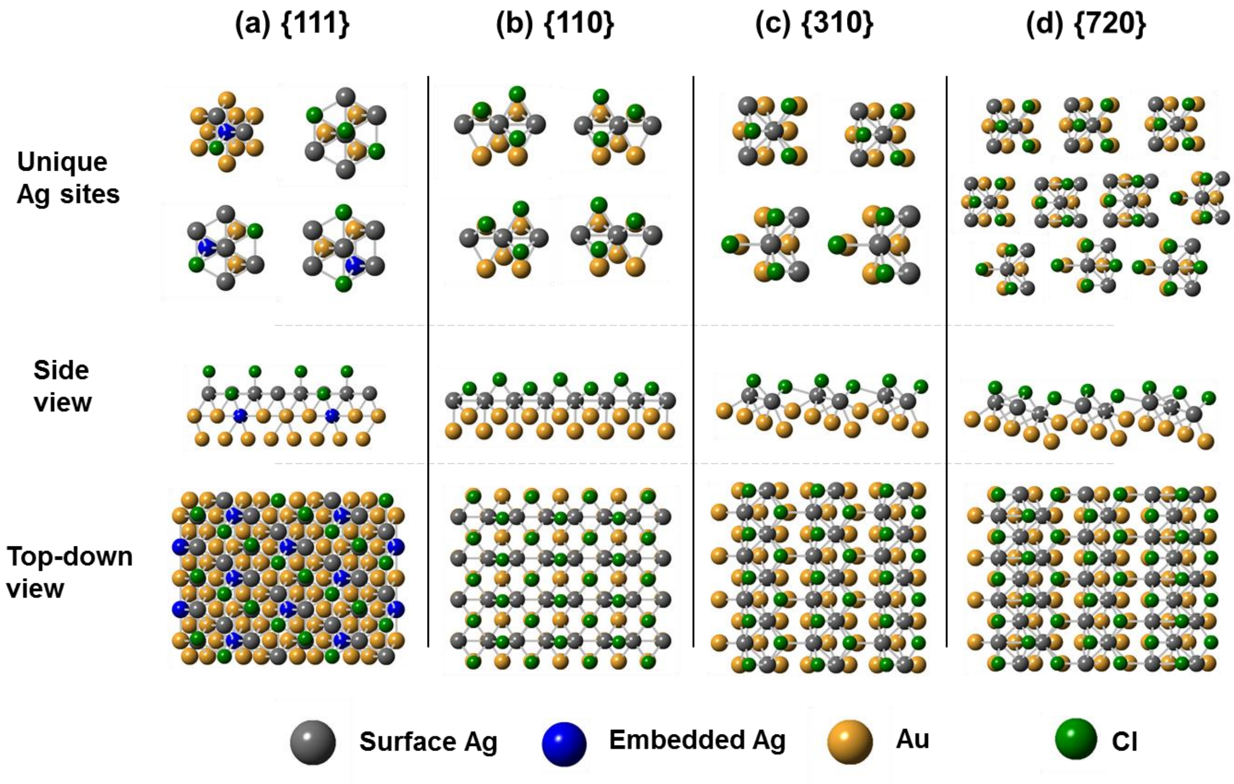


**Supplementary Figure 9. 1.0 ML $Cl^-$ DFT models.** The DFT optimized atomic coordinates of the (a) {111}, (b) {110}, (c) {310}, (d) {720} (0.20 ML) NC surfaces as modelled by Crystal Maker®. The unique Ag sites help show their individual coordination while the side and top-down views of each NC surface show overall perspective.

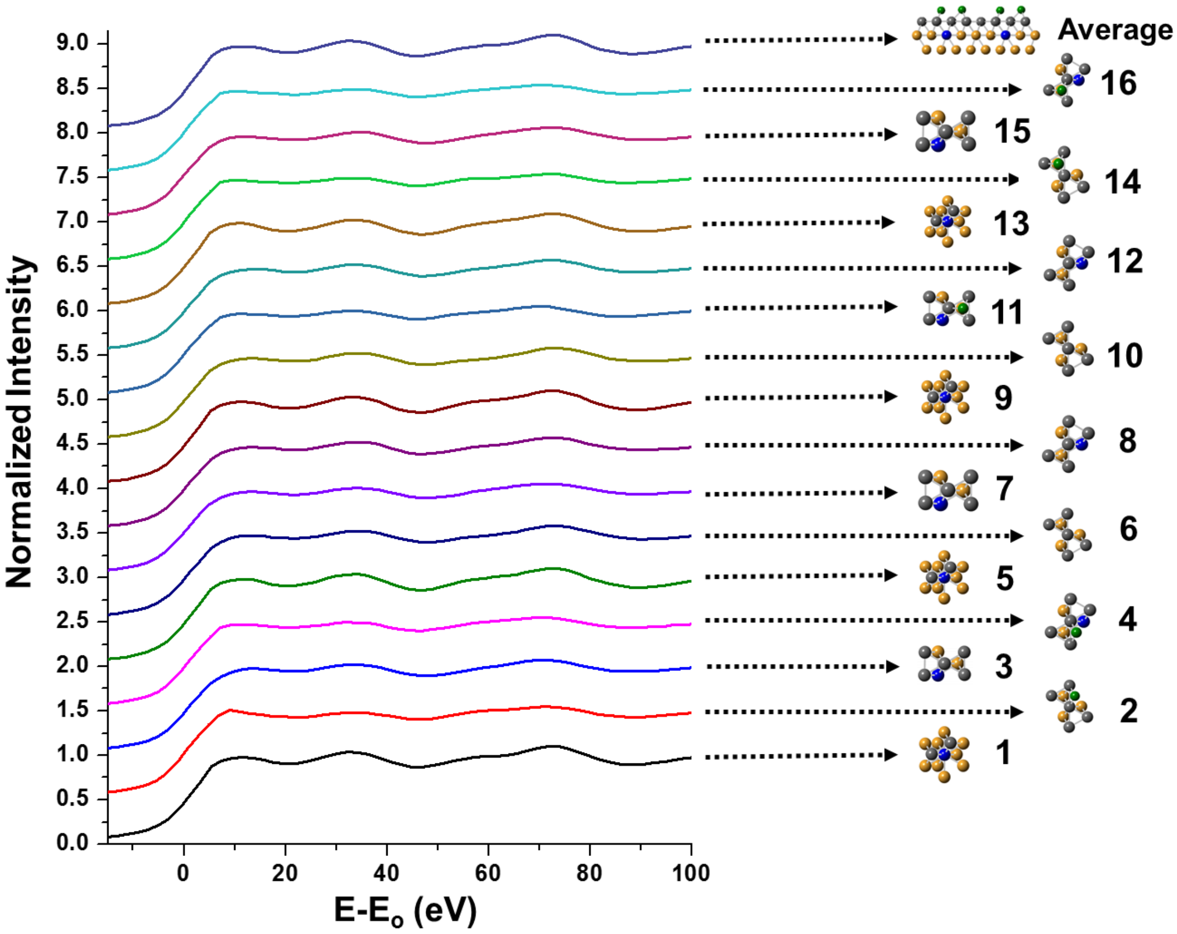


**Supplementary Figure 10. Simulated Ag K-edge XANES spectra for {111} with 0.25 ML $Cl^{-}$**.The XANES spectrum for each unique Ag site in the 0.25 ML $Cl^{-}$ {111} surface was simulated. The average of all of the spectra was presented in the main text.

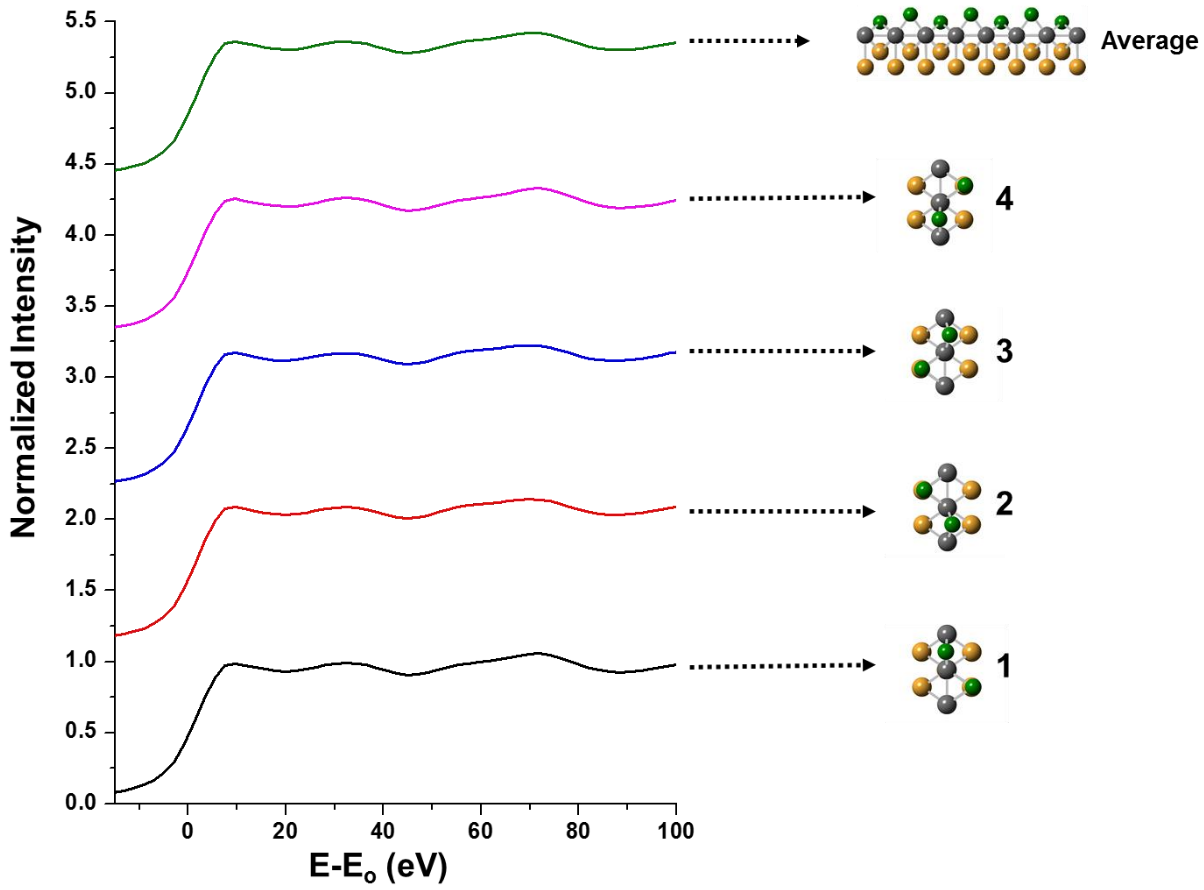


**Supplementary Figure 11. FEFF simulated Ag K-edge XANES spectra for {110} with 0.75 ML Cl$^-$**. The XANES spectrum for each unique Ag site in the 0.75 ML Cl$^-$ {110} sample was simulated. The average of all of the spectra was presented in the main text.

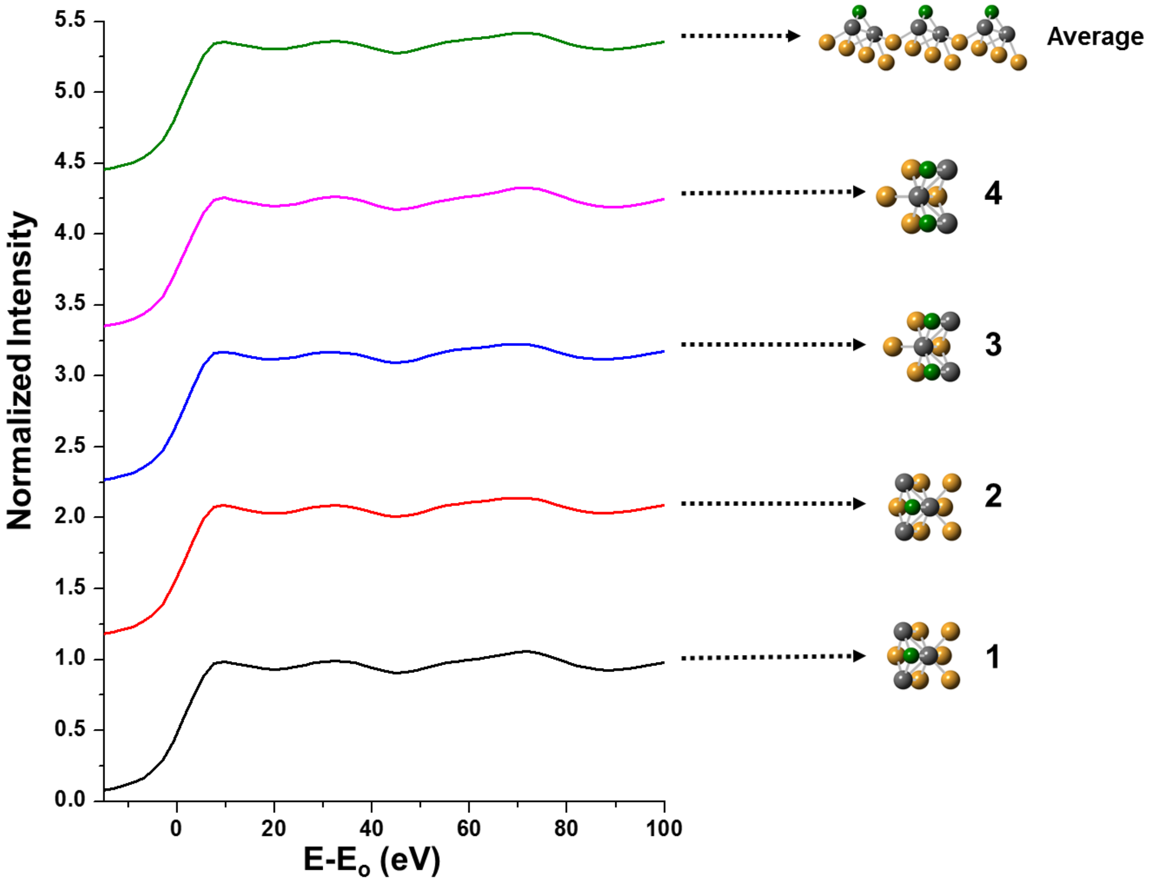


**Supplementary Figure 12. FEFF simulated Ag K-edge XANES spectra for {310} with 0.50 ML $Cl^-$.** The XANES spectrum for each unique Ag site in the 0.50 ML $Cl^-$ {310} sample was simulated. The average of all of the spectra was presented in the main text.

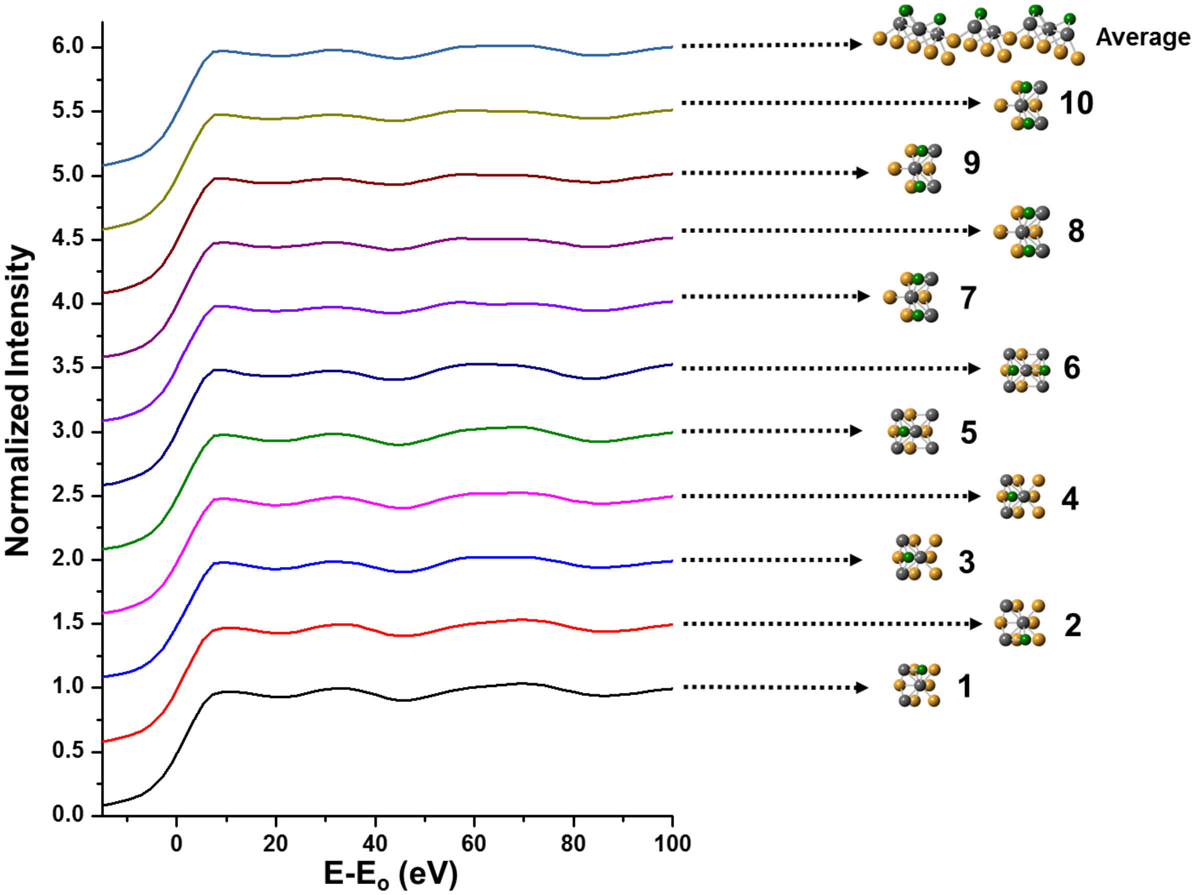


**Supplementary Figure 13. FEFF simulated Ag K-edge XANES spectra for {720} with 0.50 ML Cl$^-$.** The XANES spectrum for each unique Ag site in the 0.50 ML Cl$^-$ {720} sample was simulated. The average of all of the spectra was presented in the main text.

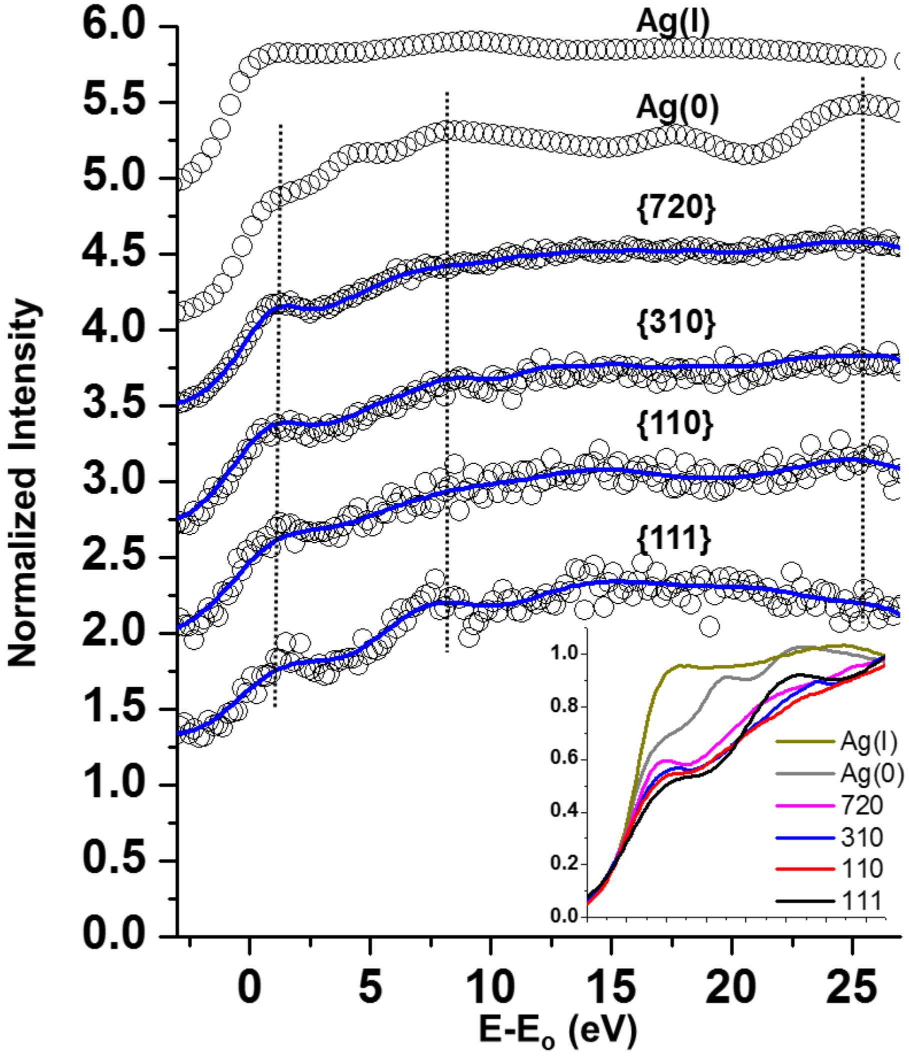


**Supplementary Figure 14. Experimental Ag $L_3$-edge XANES**. The experimental XANES of the NCs (black circles) were fit with a smooth line (in blue) in order to show overall feature similarities (black dashed lines). The large difference between the Ag(0) and Ag(I) can be seen by the overlayed spectra in the inset figure. The NCs exhibit a slightly lower intensity of the first feature which as expected for nanosized and alloyed materials. Overall, their similarity to the Ag(0) features and their peak intensities confirm their metallic state (*i.e.,* Ag(0)).

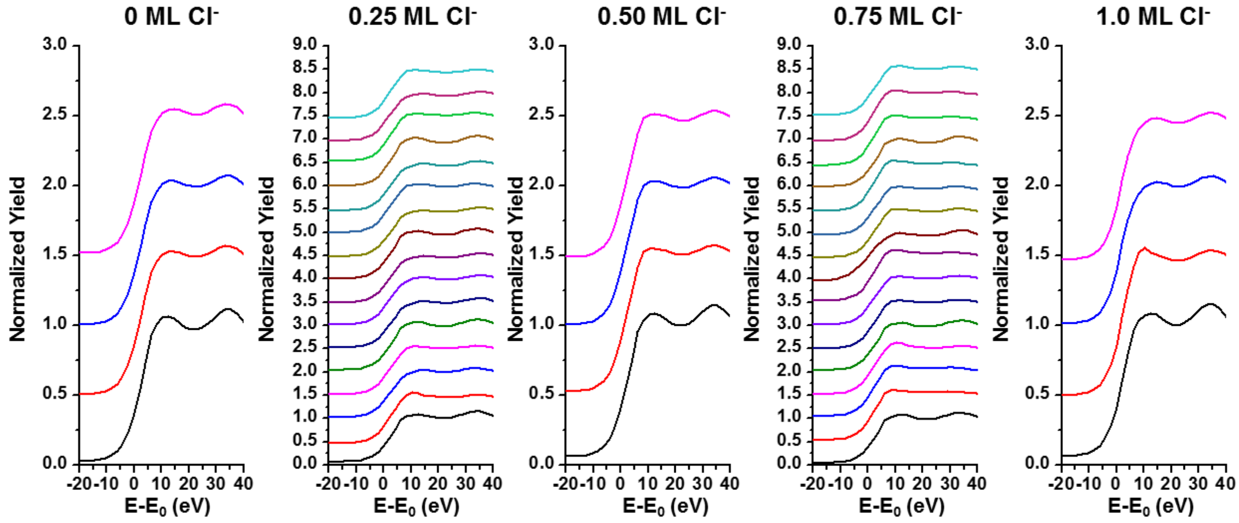


**Supplementary Figure 15. Simulated {111} Ag K-edge XANES spectra for all $Cl^-$ coverages**. The XANES spectrum for each unique Ag site (represented by different colours) in each coverage model (0, 0.25, 0.50, 0.75, and 1.0 ML $Cl^-$) was simulated by FEFF using the atomic coordinates obtained by DFT.

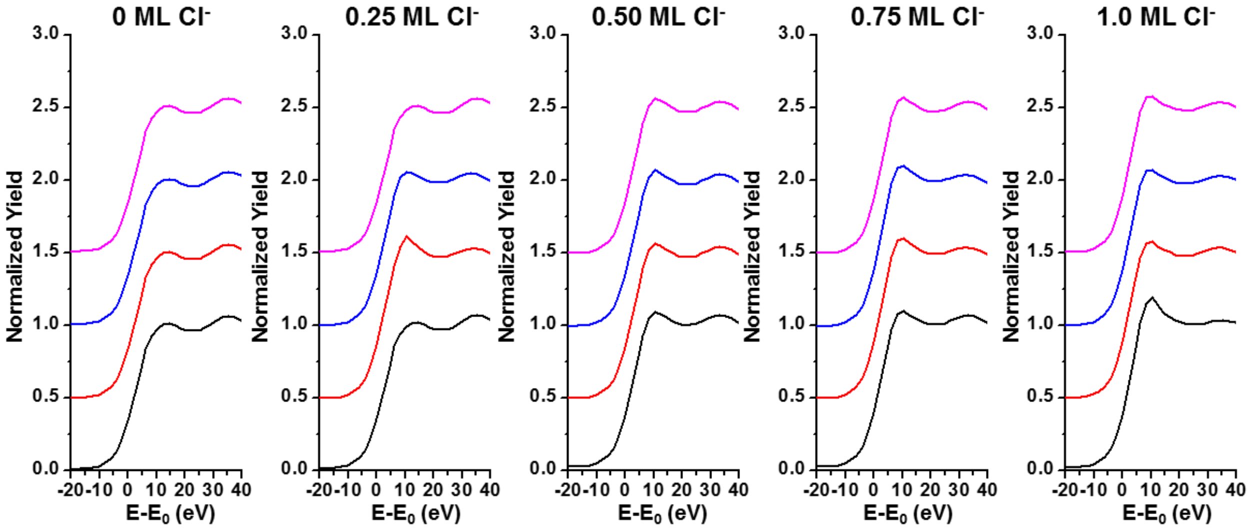


**Supplementary Figure 16. Simulated {110} Ag K-edge XANES spectra for all $Cl^-$ coverages**. The XANES spectrum for each unique Ag site (represented by different colours) in each coverage model (0, 0.25, 0.50, 0.75, and 1.0 ML $Cl^-$) was simulated by FEFF using the atomic coordinates obtained by DFT.

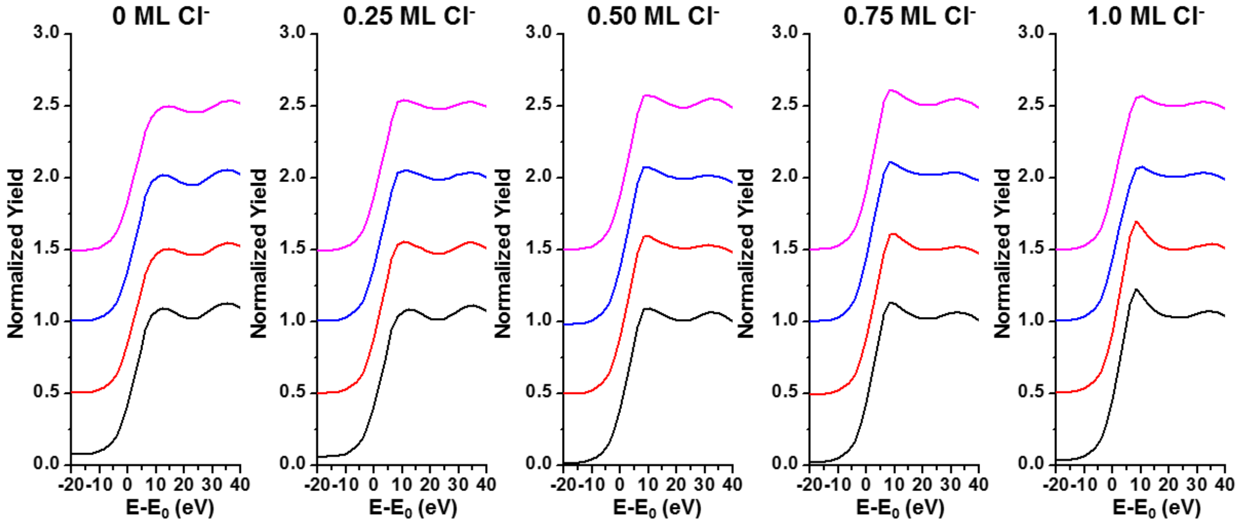


**Supplementary Figure 17. Simulated {310} Ag K-edge XANES spectra for all $Cl^-$ coverages**. The XANES spectrum for each unique Ag site (represented by different colours) in each coverage model (0, 0.25, 0.50, 0.75, and 1.0 ML $Cl^-$) was simulated by FEFF using the atomic coordinates obtained by DFT.

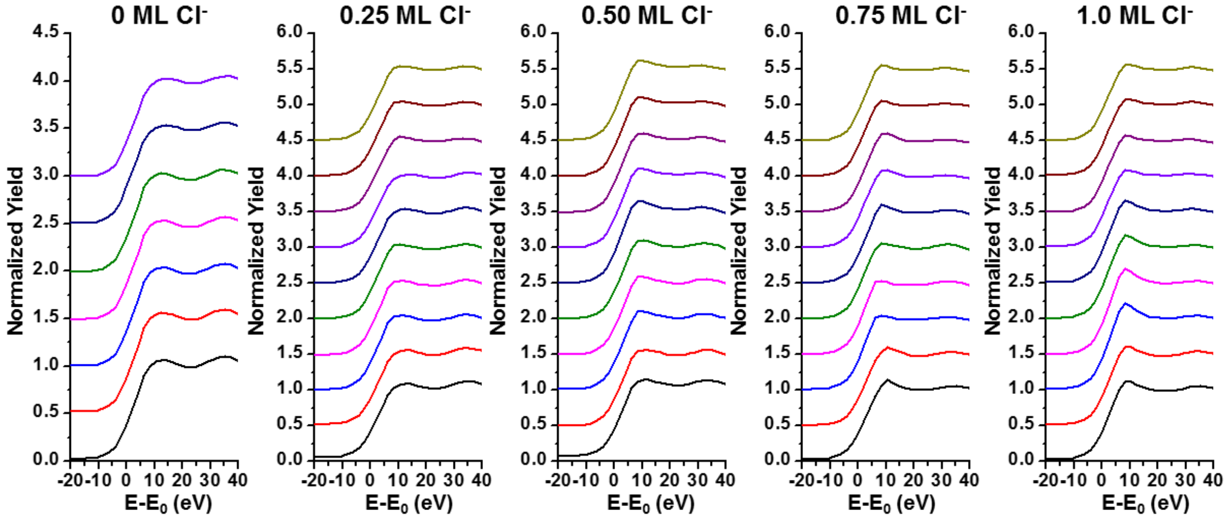


**Supplementary Figure 18. Simulated {720} Ag K-edge XANES spectra for all Cl$^-$ coverages**. The XANES spectrum for each unique Ag site (represented by different colours) in each coverage model (0, 0.25, 0.50, 0.75, and 1.0 ML Cl$^-$) was simulated by FEFF using the atomic coordinates obtained by DFT.

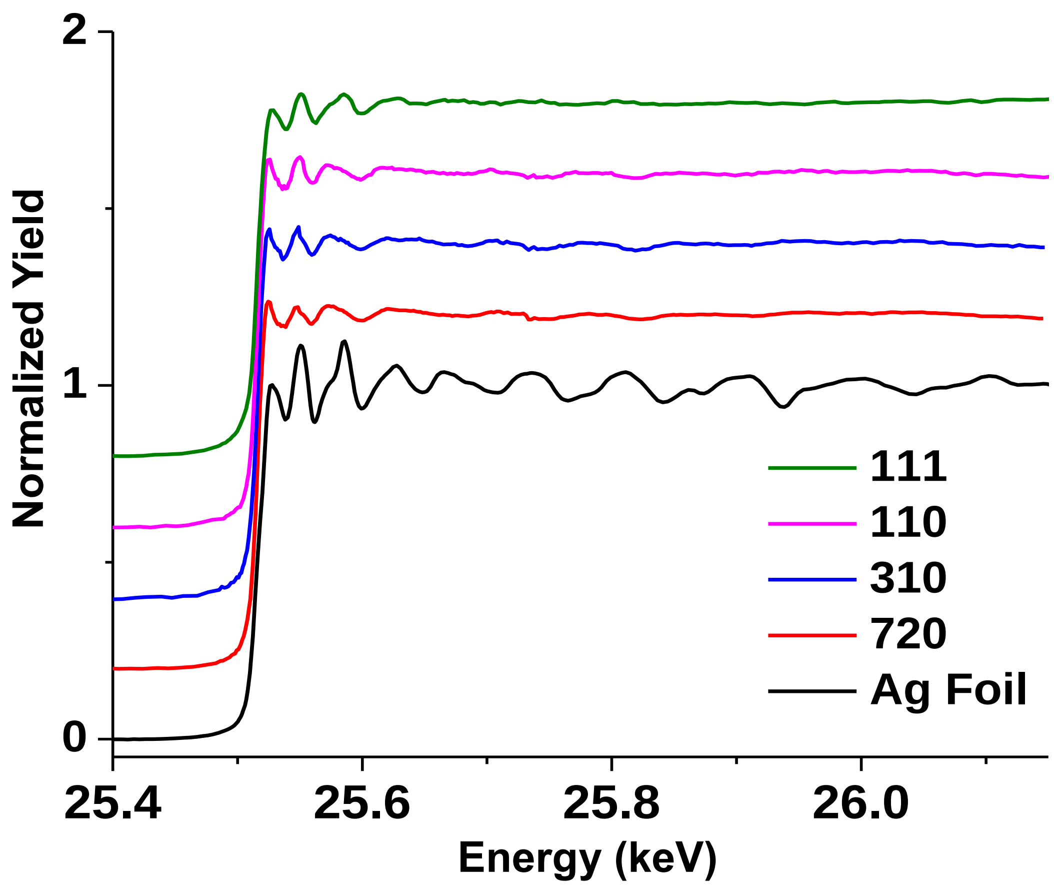


**Supplementary Figure 19. Ag K-edge EXAFS.** The Ag K-edge EXAFS for each NC surface and Ag foil was collected by X-ray Fluorescence detection at a temperature of 50 K. The spectra were background normalized and shown stacked for a qualitative comparison of oscillatory features.

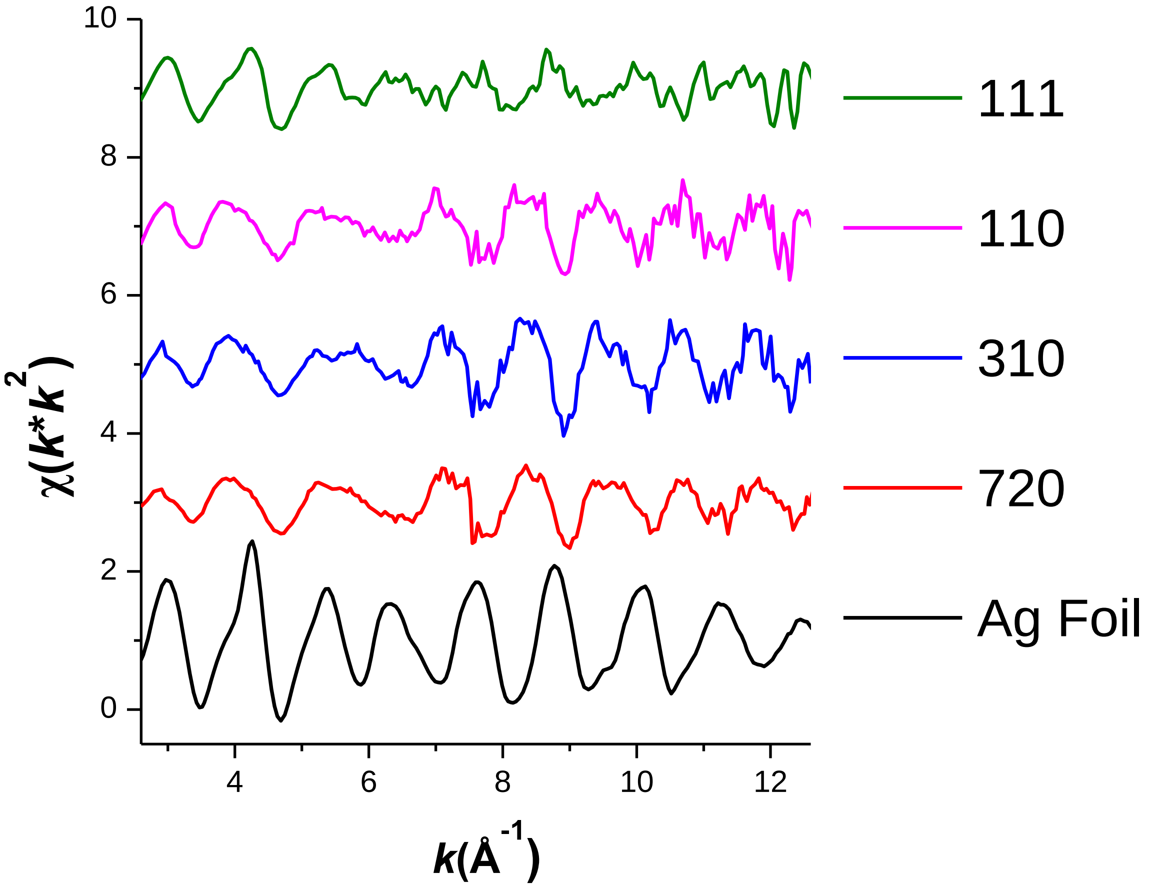


**Supplementary Figure 20. Ag K-edge EXAFS *k*-space.** The EXAFS spectra presented in Figure S19 were each fit with a spline curve to obtain *k*-space spectra presented here. The *k*-space spectra are indicative of FCC metal as seen from the oscillations similar to bulk Ag. It should be noted that the Ag bulk foil spectrum intensity was divided by a factor of 10 in order to provide a more useful comparison to the NC surfaces

**Supplementary Table 1. Ag K-edge EXAFS refinement values.** The uncertainties of the EXAFS refinement values are given by the numbers in parentheses.

| Surface | Bond | CN | R(Å) | $\sigma^2$ (Å) | $E_0$ (eV) |
|---|---|---|---|---|---|
| | Ag-Cl | 0.4(2) | 2.663(24) | 0.011(9) | -1(1) |
| **{111}** | Ag-Au | 7.1(14) | 2.991(30) | 0.021(9) | -1(1) |
| **Non-UPD** | Ag-Ag | 3.4(3) | 2.878(6) | 0.009(1) | -1(1) |
| | Ag-Cl | 1.8(4) | 2.624(8) | 0.009(3) | -7(1) |
| **{110}** | Ag-Au | 6.8(7) | 2.870(4) | 0.007(1) | -7(1) |
| **UPD** | Ag-Ag | 0.8(1) | 2.790(10) | 0.007(1) | -7(1) |
| | Ag-Cl | 1.2(2) | 2.611(9) | 0.006(2) | -6(1) |
| **{310}** | Ag-Au | 4.9(8) | 2.880(8) | 0.004(1) | -6(1) |
| **UPD** | Ag-Ag | 0.6(1) | 2.887(30) | 0.004(1) | -6(1) |
| | Ag-Cl | 1.5(4) | 2.587(10) | 0.007(3) | -6(1) |
| **{720}** | Ag-Au | 5.7(9) | 2.863(10) | 0.006(1) | -6(1) |
| **UPD** | Ag-Ag | 0.6(1) | 2.842(50) | 0.006(1) | -6(1) |

**Supplementary Table 2. DFT versus EXAFS CN results.** The DFT CNs for each coverage are shown (background darkened to distinguish DFT results), while the proposed surface models based on the experimental results are highlighted in red. The uncertainties of the EXAFS refinement values are given by the numbers in parentheses.

| Surface | Bond | EXAFS CN | 0 ML | 0.25 ML | 0.5 ML | 0.75 ML | 1.0 ML |
|---|---|---|---|---|---|---|---|
| **{111}** | Ag-Cl | 0.4(2) | - | 0.3 | 0.75 | 1.1 | 2 |
| **Non-UPD** | Ag-Au | 7.1(14) | 4 | 4 | 4 | 4 | 4 |
| | Ag-Ag | 3.4(3) | 3.25 | 3.6 | 4 | 4 | 4 |
| **{110}** | Ag-Cl | 1.8(4) | - | 0.5 | 1 | 2 | 2.5 |
| **UPD** | Ag-Au | 6.8(7) | 5 | 5 | 5 | 5 | 5 |
| | Ag-Ag | 0.8(1) | 2 | 2 | 2 | 2 | 2 |
| **{310}** | Ag-Cl | 1.2(2) | - | 0.75 | 1.5 | 2 | 3 |
| **UPD** | Ag-Au | 4.9(8) | 5 | 5 | 5 | 5 | 5 |
| | Ag-Ag | 0.6(1) | 2 | 2 | 2 | 2 | 2 |
| **{720}** | Ag-Cl | 1.5(4) | - | 0.6 | 1.4 | 2.5 | 3.2 |
| **UPD** | Ag-Au | 5.7(9) | 4.8 | 4.8 | 4.8 | 4.8 | 4.8 |
| | Ag-Ag | 0.6(1) | 3.0 | 2.2 | 2.2 | 2.2 | 2.2 |
